# BuPiHeWei Decoction Ameliorates 5-Fu-Induced Intestinal Mucosal Injury in the Rats by Regulating the TLR-4/NF-*κ*B Signaling Pathway

**DOI:** 10.1155/2019/5673272

**Published:** 2019-12-19

**Authors:** Zhi-gao Sun, Ya-zhuo Hu, Yu-guo Wang, Jian Feng, Yong-qi Dou

**Affiliations:** ^1^Department of Traditional Chinese Medicine, Chinese PLA General Hospital, Beijing 100853, China; ^2^Department of Traditional Chinese Medicine, Hainan Hospital of Chinese PLA General Hospital, Sanya 572004, Hainan Province, China; ^3^Institute of Gerontology, Chinese PLA General Hospital, Beijing 100853, China

## Abstract

BuPiHeWei (BPHW) decoction, a classic Traditional Chinese Medicinal (TCM) prescription, has been widely used in clinical practice to relieve digestive symptoms caused by chemotherapy, such as diarrhea and vomiting. The present study aimed to investigate whether BPHW decoction exerted a protective role in the 5-Fu-induced intestinal mucosal injury in the rats by regulating the mechanisms of TLR-4/NF-*κ*B signaling pathway. There were 35 Sprague Dawley rats randomly divided into four groups: normal control group, 5-Fu group, 5-Fu + BPHW decoction group (10.5 g/kg, for five continuous days), and 5-Fu + *Bacillus licheniformis* capsule group (0.2 g/kg, for five continuous days). Animal models were established by intraperitoneal injection of 5-Fu (30 mg/Kg, for five consecutive days). At the end of the treatment period, body weight, diarrhea score, and histological examination were examined. Furthermore, the expression of TLR-4/NF-*κ*B pathway was detected to reveal its mechanism. The results showed that BPHW decoction effectively reduced diarrhea score and increased body weight and height of villi after 5-Fu chemotherapy. In addition, BPHW decoction could significantly inhibit the expression of TLR-4, NF-*κ*B, and inflammatory factors (including TNF-*α*, IL-1*β*, and IL-6) in the intestine, and the efficacy was significantly higher than that of *Bacillus licheniformis* capsule. In summary, BPHW decoction might be considered an effective drug to alleviate intestinal mucosal injury in the rats induced by 5-Fu.

## 1. Introduction

The intestinal toxicity caused by chemotherapy is mainly characterized by the destruction of intestinal mucosal barrier and bacterial flora disorder. In severe cases, bacterial translocation might occur to cause life-threatening bacteremia. Despite the continuous development and application of mucosal protective agents and microecologics, the incidence of adverse reactions after chemotherapy, including diarrhea, abdominal pain, and constipation, is still more than 80% [1]. 5-Fu and irinotecan are considered as having obvious gastrointestinal toxicity caused by chemotherapy, which is closely associated with intestinal mucosal microinflammation [2]. Previous studies [3] have shown that physical and chemical factors stimulate intestinal microinflammation through five stages: initiation, induction, multiplication, ulceration, and healing and induce the apoptosis of intestinal mucosal cells and cell cycle disorder through p53 mechanism. Subsequently, on the one hand, it leads to the remodeling of tight junction-associated proteins and reducing the depth of the crypt; on the other hand, it hinders cell regeneration and intestinal mucosal repair, ultimately destructing barrier function. In addition, the pathogenesis of chemotherapy-associated intestinal inflammation is complicated, involving various factors, such as immunity, apoptosis, and dysbacteriosis. In recent years, toll-like receptor (TLR)/NF-*κ*B pathway has been found as the focal point and core mechanism of multiple signal transduction pathways, finally inducing the cascade-like release of proinflammatory cytokines, such as TNF-*α*, IL-6, and IL-1*β*, and causing an uncontrolled inflammatory response [4].

Clinical studies have shown that BPHW decoction can ameliorate chemotherapy-induced gastrointestinal symptoms, such as nausea, vomiting, bloating, diarrhea, and anorexia. Relevant studies [5] have also confirmed that it harbors biological activities, including immunity regulation, anti-inflammation, and antioxidation. However, its molecular biological mechanism remains unclear. This study was designed to explore whether BPHW decoction exerted a protective role in chemotherapy-induced intestinal mucosal injury and whether its mechanisms were related to the TLR-4/NF-*κ*B signaling pathway.

## 2. Materials and Methods

### 2.1. Rats

Thirty-five male Sprague Dawley rats (200 ± 10 g, 6 weeks) were purchased from the Experimental Animal Center of the 302 Military Hospital (animal certificate number: SYXK (Army)-2012-0010). All rats were housed on a 12 h light/dark cycle in a temperature- and humidity-controlled room and maintained on a standard diet and water ad libitum. Animal experimental procedures were performed in accordance with the Guidance Suggestions for the Care and Use of Laboratory Animals, formulated by the Ministry of Science and Technology of China, and the protocols were approved by the Animal Ethics Committee at Chinese PLA General Hospital (Beijing, China).

### 2.2. Preparation of BPHW Decoction and *Bacillus licheniformis* Solution

BPHW Decoction was composed of astragalus (20 g), *Atractylodes* (15 g), clamshell (10 g), ginger pinellia (10 g), divine song (15 g), malt (15 g), and alfalfa (15 g). Herbs were provided and identified by the Chinese Pharmacy of Chinese PLA General Hospital (Beijing, China). In brief, the medicine was mixed, added to 1.5-fold volume of water, decocted, and filtered, followed by the concentration at a constant temperature water bath at 80°C to a crude drug containing 5 g/ml of drug solution, which was subsequently stored at 4°C for further application.

*Bacillus licheniformis* capsule was purchased from Northeast Pharmaceutical of China (product lot number: S10950019, specification: 0.25 g/grain), and 0.2 g/kg *Bacillus licheniformis* capsule physiological saline was prepared for use. The above drugs were converted according to the clinical dose for adults.

### 2.3. Reagents and Instruments

Anti-ZO-1, anti-E-Cadherin, anti-TLR-4, and anti-NF-*κ*B antibodies were purchased from Abcam (Cambridge Science Park in Cambridge, UK). TNF-*α*, IL-1*β*, and IL-6 enzyme-linked immunosorbent assay (ELISA) kits were obtained from Expandbiotech (Life Science Park, Beijing, China). Real-time quantitative PCR kit LightCycler® 480 SYBR Green I Master (cat. no. 04707516001, USA) was purchased from Roche Applied Science (Basel, Swiss).

Real-time quantitative PCR instrument (Science 384, Applied Basel, Swiss), microplate reader (Multiskcan FC, Applied Thermo Fisher, USA), and electrophoresis apparatus (PowerPac Basic, Applied Bio-Rad, USA) were used in the study.

### 2.4. Model Establishment and Treatment

Random number method was used to divide all rats into blank control group (N, *n* = 8), model group (M, *n* = 9), 5-Fu + BPHW decoction group (BPHW, *n* = 9), and 5-Fu + *Bacillus licheniformis* capsule group (BLC, *n* = 9). Rats in the blank control group were intraperitoneally injected with the same volume of normal saline, and rats in all the other groups were intraperitoneally injected with 5-Fu for five continuous days with 30 mg/Kg/d (10 ml: 0.25 g Shanghai Xudong Haipu Pharmaceutical Co., Ltd.) to establish the chemotherapeutic model.

BPHW decoction and *Bacillus licheniformis* capsule were converted according to the equivalent dose of adults. BPHW decoction was given at 10.5 g/kg daily for five continuous days at the beginning of model establishment. *Bacillus licheniformis* capsule was given by gavage at 0.2 g/kg in 2 ml of normal saline daily for five continuous days at the beginning of model establishment. Twenty-four hours after gavage, all rats in each group were executed with spinal dislocation. Small intestine (jejunum) tissues were removed, partially fixed in 4% paraformaldehyde, and the remaining part was stored at −80°C in a refrigerator for further analysis. Finally, five specimens in each group were randomly selected for testing.

### 2.5. Body Weight and Diarrhea Score of Rats in Each Group

On the 0th, 3rd, and 6th day, body weight of rats was measured, and the stool quality was observed in each group. Diarrhea was scored according to the AKinobu Kurital score [6] (Table 1).

### 2.6. Histological Examination

The jejunum tissue was fixed in 4% paraformaldehyde for 24 h at 4°C and then dehydrated, embedded in paraffin, and sliced in cryostat (4 mm thick). The pathological changes of jejunum were observed under light microscope after hematoxylin and eosin (H&E) staining.

### 2.7. Western Blot Evaluation for ZO-1, E-Cadherin, TLR-4, and NF-*κ*B of Jejunum Mucosa

Total protein was extracted according to the relevant instructions, and BCA method was used for protein quantitation. The supernatant from the above lysate (containing about 80 *μ*g of protein) was subjected to 12% SDS-PAGE and transferred onto a nitrocellulose (NC) membrane. The membranes were probed with primary antibodies against ZO-1, E-cadherin, TLR-4, NF-*κ*B, and *β*-actin followed by incubation with secondary antibodies conjugated with horseradish peroxidase (HRP, Thermo Fisher, Waltham, MA, USA). Millipore ECL (Millipore, Massachusetts, USA) was used for chemiluminescence, film exposure, and fixation, and grayscale value was analyzed by using Image Lab software (BioRad, Hercules, CA, USA).

### 2.8. Immunohistochemistry

The jejunum tissue was fixed in 4% paraformaldehyde for 24 h at 4°C, embedded in paraffin, and sectioned for 4 mm thickness. The sections are then heated in a constant temperature oven (60°C) for 4 h, deparaffinized with xylene, and hydrated by 100%, 95%, and 85% graded ethanol. The sections were washed with PBS, blocked with 3% H_2_O_2_ for 10 min, and heated in citrate buffer (pH 6.0) in a microwave. The sections were washed with PBS again, incubated with TLR-4 polyclonal antibody (1 : 200 dilution), NF-*κ*B polyclonal antibody (1 : 300 dilution) at 4°C, overnight, and the negative control group was incubated in PBS (0.01 mol/L) instead of the primary antibody. The following day, after being washed with PBS for 3 times, the sections were incubated with biotinylated secondary antibodies for 30 min and then stained with DAB and lightly counterstained with hematoxylin. Finally, after being hydrated by 85%, 95%, and 100% graded ethanol, the sections were sealed with neutral gum and then observed under the microscope. The Image-Pro Plus 5.1 image analytical system was used to measure the integrated optical density (IOD).

### 2.9. QPCR Evaluation for TLR-4 and NF-*κ*B of Jejunum Mucosa

Primer 5.0 software was used for primer design according to the gene sequence in Genbank database (Table 2). Total RNA was extracted according to the kit manufacturer's instruction, followed by determination of RNA concentration and purification by using the UV spectrophotometer (NanoDrop2000, Thermo Scientific). RNA was reversely transcribed into cDNA in line with relevant protocol. The PCR reaction mixture (10 *μ*L) consisted of 5 *μ*L 2 × Master Mix (PCR enzyme), 0.5 *μ*L forward primer, 0.5 *μ*L reverse primer, 1 *μ*L cDNA template, and 3 *μ*L RNase-free water. Two-step PCR reaction was employed by using the following conditions: 95°C denaturation for 5 min, 40 cycles under the following conditions: denaturation at 95°C for 10 s, annealing at 55°C for 15 s, and extension at 72°C for 20 s. The SYBR green fluorescent signals were acquired at 72°C. Standard curves were constructed from PCR reactions using 10-fold serial dilutions of known bacterial DNA. The relative gene expression levels were converted, normalized, and expressed as fold changes (=2^−ΔΔCt^).

### 2.10. ELISA Assay for TNF-*α*, IL-1*β*, and IL-6 in Jejunum Mucosa

The levels of TNF-*α*, IL-1*β*, and IL-6 in the jejunum tissues were examined by ELISA according to the kit instructions. The optical density values were measured at 450 nm using a microplate spectrophotometer, and the results were calculated from the standard curve.

### 2.11. Statistical Analysis

SPSS17.0 software package (SPSS, Chicago, IL, USA) was used for statistical analysis. Variance analysis was performed for normally distributed and homogeneous measurement data, which were shown as $\bar{x}\pm s$. Rank sum test was used if the data were not normally distributed or heterogeneous. A *P* < 0.05 was considered as statistical significance.

## 3. Results

### 3.1. The Effects of BPHW Decoction on Body Weight and Diarrhea Score in the Rats Induced by 5-Fu Chemotherapy

**Figure 1:**
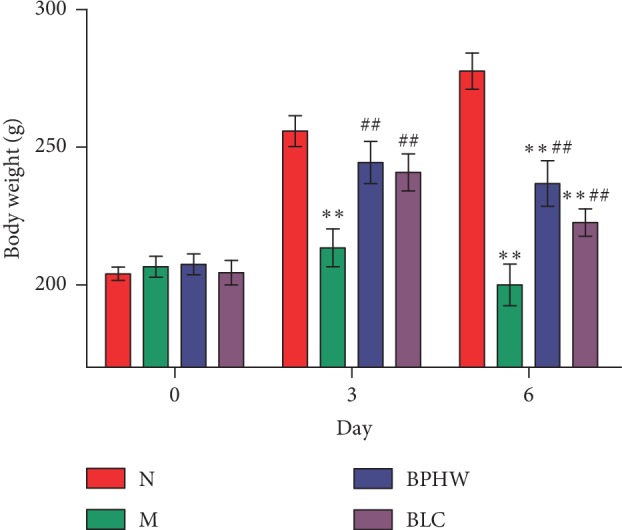
Comparison of body weight among different groups ($\bar{x}\pm s$, *n* = 8/9). ^*∗∗*^*P* < 0.01 versus the normal group. ^ΔΔ^*P* < 0.01 versus the model group.

There was no significant difference in body weight among different groups before the experiment (*P* > 0.05). The body weight of rats in the blank control group was naturally increased during the experiment. Compared with the blank control group, body weight of rats in the model group was significantly decreased (*P* < 0.01). In comparison with the model group, body weight of rats in both BPHW decoction group and *Bacillus licheniformis* capsule group were significantly increased during the chemotherapy period (*P* < 0.01). In spite of the absence of statistical significance, average body weight of rats in the BPHW decoction group was higher than that in the *Bacillus licheniformis* capsule group (Figure 1).

**Figure 2:**
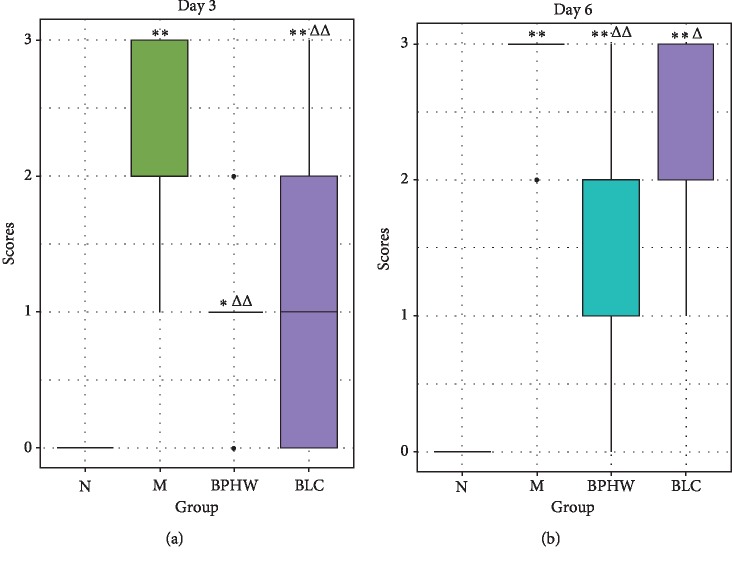
Comparison of diarrhea scores among different groups ($\bar{x}\pm s$, *n* = 8/9). ^*∗*^*P* < 0.05 and ^*∗∗*^*P* < 0.01 versus the normal group. ^Δ^*P* < 0.05 and ^ΔΔ^*P* < 0.01 versus the model group.

The diarrhea score of rats was 0 in all the groups before the experiment. There was no diarrhea in the rats of the blank control group throughout the experiment. In contrast, the diarrhea score was significantly increased on the 3rd and 6th days in the model group, showing a progressively aggravating trend (*P* < 0.01). Compared with those in the model group, the diarrhea scores of rats were significantly lower in both BPHW decoction group and *Bacillus licheniformis* capsule group (*P* < 0.01, *P* < 0.05). Although there was no statistical difference, the diarrhea score was lower in the rats of the BPHW decoction group than that in the *Bacillus licheniformis* capsule group (Figure 2).

### 3.2. The Effects of BPHW Decoction on Intestinal Mucosal Integrity of 5-Fu Chemotherapy Rats

**Figure 3:**
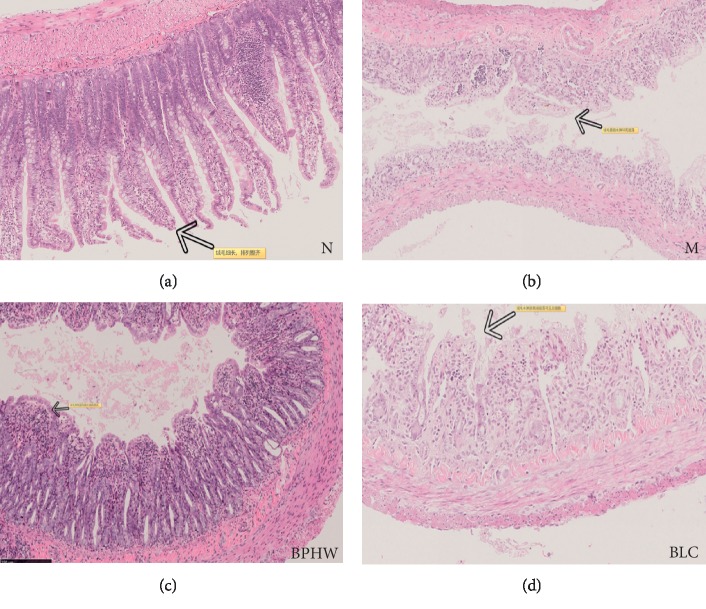
Effects of BPHW decoction on jejunum histological examination (*n* = 5, H&E stain, magnification ×10).

HE staining showed that the intestinal mucosa of rats in the blank control group was intact, with regularly arranged glands and without obvious histopathological changes. The intestinal villi in rats of the model group were shed, with edema, degeneration, and necrosis, infiltrated with inflammatory cells. These pathological alterations were alleviated in the intestinal mucosa of rats in both BPHW decoction group and *Bacillus licheniformis* capsule group, which were more obvious in the BPHW decoction group (Figure 3).

### 3.3. The Effects of BPHW Decoction on the Protein Expression of ZO-1, E-Cadherin, TLR-4, and NF-*κ*B in Intestinal Mucosa of Rats with 5-Fu Chemotherapy

**Figure 4:**
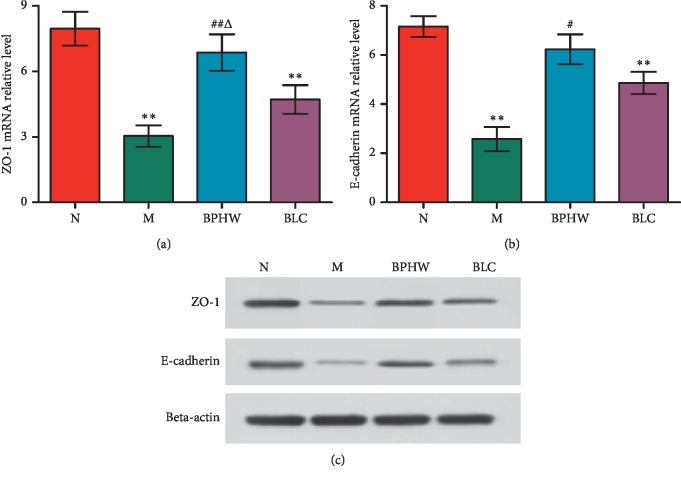
(a–c) The protein expression of ZO-1 and E-cadherin in the jejunum of rats in each group ($\bar{x}\pm s$, *n* = 5). ^*∗∗*^*P* < 0.01 versus the normal group. ^#^*P* < 0.05 and ^##^*P* < 0.01 versus the model group. ^Δ^*P* < 0.05 and ^ΔΔ^*P* < 0.01 versus the BLC group.

Western blot showed that compared with the blank control group, the protein expression of ZO-1 and E-cadherin in the jejunum of rats in the model group and the *Bacillus licheniformis* capsule group were significantly decreased (*P* < 0.01), while that in the BPHW decoction group was not statistically altered (*P* > 0.05). The protein expression of ZO-1 and E-cadherin in rats of the BPHW decoction group was significantly increased than that in the model group (*P* < 0.01 and *P* < 0.05), and the protein expression of ZO-1 in rats of the BPHW decoction group was significantly increased than that in the *Bacillus licheniformis* capsule group (*P* < 0.05) (Figures 4(a)–4(c)).

**Figure 5:**
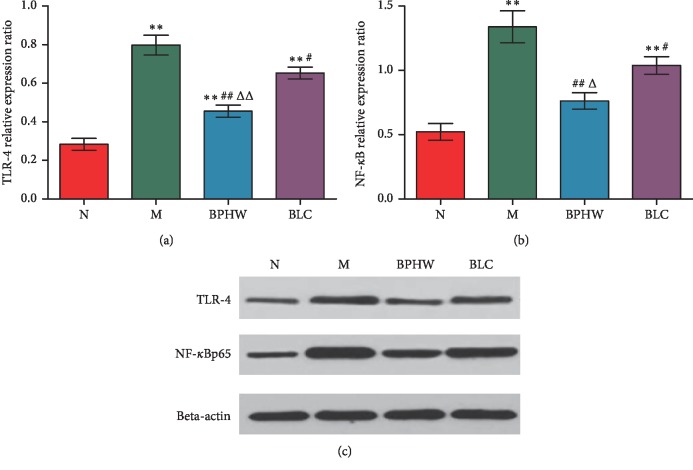
(a–c) The protein expression of TLR-4 and NF-*κ*B in the jejunum of rats in each group ($\bar{x}\pm s$, *n* = 5). ^*∗∗*^*P* < 0.01 versus the normal group. ^#^*P* < 0.05 and ^##^*P* < 0.01 versus the model group. ^Δ^*P* < 0.05 and ^ΔΔ^*P* < 0.01 versus the BLC group.

On the contrary, compared with the blank control group, the protein expression of TLR-4 and NF-*κ*B in the jejunum of rats in the model group and the *Bacillus licheniformis* capsule group were significantly increased (*P* < 0.01). In addition, the protein expression of TLR-4 was significantly increased (*P* < 0.01), while that of NF-*κ*B was not statistically different (*P* > 0.05) in rats of the BPHW decoction group, in comparison with those in the blank control group. The protein expression of TLR-4 and NF-*κ*B was significantly decreased in rats of both BPHW group and *Bacillus licheniformis* capsule group (*P* < 0.01, *P* < 0.05) compared with the model group. Moreover, the protein expression of TLR-4 and NF-*κ*B was significantly decreased in rats of the BPHW group than that of the *Bacillus licheniformis* capsule group (*P* < 0.01 and *P* < 0.05) (Figures 5(a)–5(c)).

### 3.4. The Effects of BPHW Decoction on the Expression of TLR-4 and NF-*κ*B in the Jejunum of 5-Fu-Simulated Chemotherapeutic Rats

**Figure 6:**
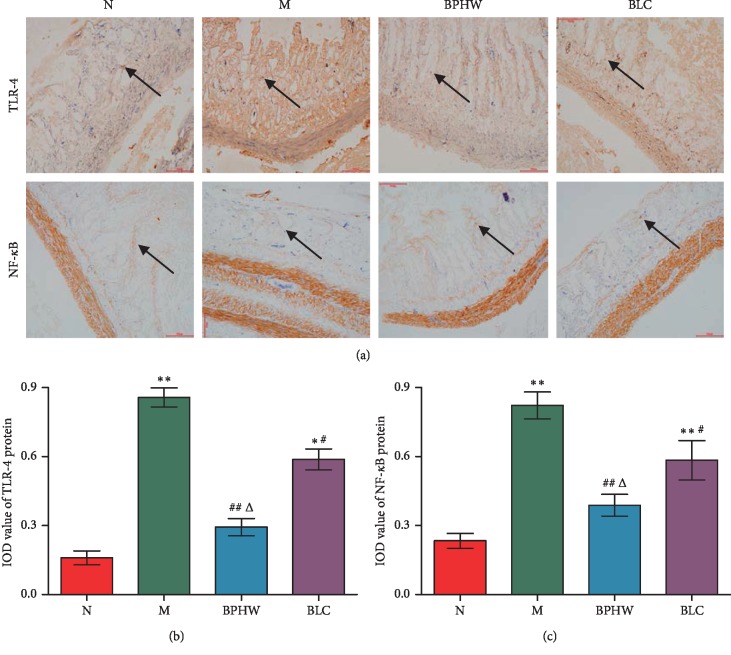
The expression of TLR-4 and NF-*κ*B in the jejunum of rats in each group ($\bar{x}\pm s$, *n* = 5). (a) Immunohistochemistry stain (magnification ×200). IOD value of (b) TLR-4 protein expression and (c) NF-*κ*B protein expression. ^*∗*^*P* < 0.05 and ^*∗∗*^*P* < 0.01 versus the normal group. ^#^*P* < 0.05 and ^##^*P* < 0.01 versus the model group. ^Δ^*P* < 0.05 versus the BLC group.

As shown in Figure 6, the positive expression of TLR-4 and NF-*κ*B protein was stained in brown, and a few of them were observed in jejunum tissue of the normal group. In contrast, TLR-4 and NF-*κ*B expression was significantly higher in the jejunum of the model group (*P* < 0.01). Compared with the model group, the positive products in the jejunum were reduced with BPHW and BLC treatment (*P* < 0.01, *P* < 0.05), and the expression of TLR-4 and NF-*κ*B in the BPHW group was lower than that in the BLC group (*P* < 0.05).

### 3.5. The Effects of BPHW Decoction on the mRNA Expression of TLR-4 and NF-*κ*B in Intestinal Mucosa in Rats with 5-Fu Chemotherapy

**Figure 7:**
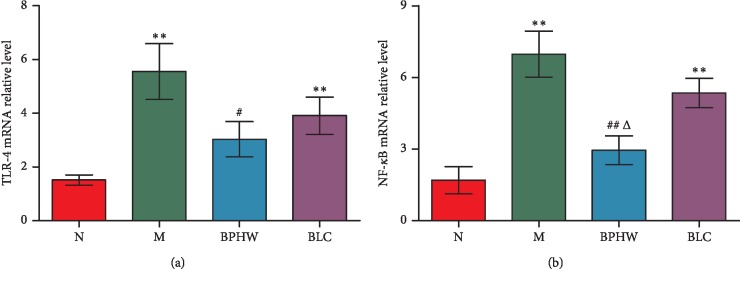
The mRNA expression of (a) TLR-4 and (b) NF-*κ*B in the jejunum of rats in each group ($\bar{x}\pm s$, *n* = 5). ^*∗*^*P* < 0.05 and ^*∗∗*^*P* < 0.01 versus the normal group. ^#^*P* < 0.05 and ^##^*P* < 0.01 versus the model group. ^Δ^*P* < 0.05 versus the BLC group.

QPCR showed that in comparison with the blank control group, the mRNA expression of TLR-4 and NF-*κ*B in the jejunum of rats in the model group and the *Bacillus licheniformis* capsule group were significantly increased (*P* < 0.01), while that in the BPHW decoction group was not statistically altered (*P* > 0.05). The mRNA expression of TLR-4 and NF-*κ*B in rats of the BPHW decoction group was significantly decreased than that in the model group (*P* < 0.05, *P* < 0.01), while there was no significant difference between the *Bacillus licheniformis* capsule group and the model group (*P* > 0.05). The mRNA expression of NF-*κ*B in rats of the BPHW decoction group was significantly lower than that in the *Bacillus licheniformis* capsule group (*P* < 0.05), while there was no statistical significance in the mRNA expression of TLR-4 between the two groups (*P* > 0.05) (Figures 7(a) and 7(b)).

### 3.6. The Effects of BPHW Decoction on the Contents of TNF-*α*, IL-1*β*, and IL-6 in Intestinal Mucosa of Rats with 5-Fu Chemotherapy

**Figure 8:**
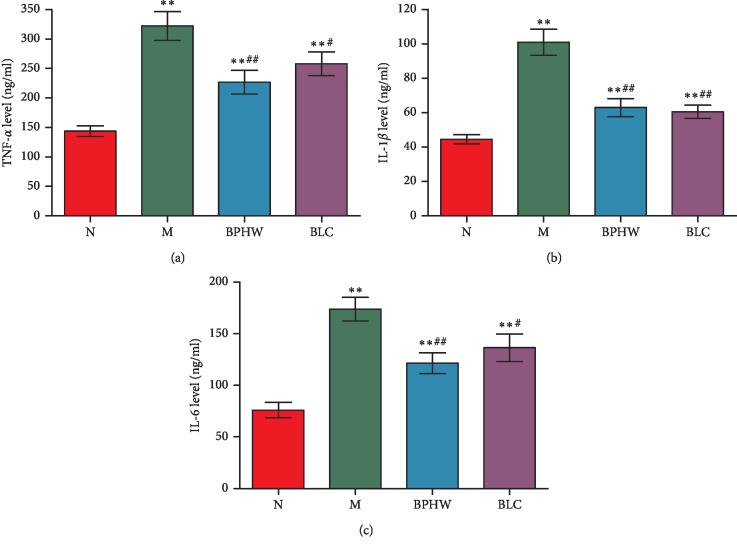
The expression of (a) TNF-*α*, (b) IL-1*β*, and (c) IL-6 in the jejunum of rats in each group ($\bar{x}\pm s$, *n* = 5). ^*∗∗*^*P* < 0.01 versus the normal group. ^#^*P* < 0.05 and ^##^*P* < 0.01 versus the model group.

ELISA assay showed the contents of TNF-*α*, IL-1*β*, and IL-6 in the jejunum of rats in the model group, BPHW decoction group, and *Bacillus licheniformis* capsule group were significantly increased (*P* < 0.01) compared with the blank control group. In addition, the contents of TNF-*α*, IL-1*β*, and IL-6 was significantly decreased in rats of the BPHW decoction group and *Bacillus licheniformis* capsule group in comparison with the model group (*P* < 0.01 and *P* < 0.05). There was no statistical significance between the BPHW decoction group and *Bacillus licheniformis* capsule group (*P* > 0.05) (Figures 8(a)–8(c)).

## 4. Discussion

Diarrhea and weight loss are common side effects of chemotherapy, which are associated with the abnormality of ion transport in the lamina propria and the decreased absorption of water and electrolyte due to chemotherapy-induced intestinal mucosa and villus damage [7, 8]. Symptomatic therapeutic drugs, such as intestinal microecologics (dominated by prebiotics), have constantly emerged in recent years. However, the incidence of intestinal side effects caused by chemotherapy has not been significantly decreased [9]. Traditional Chinese Medicine believes that “spleen governs transportation and transformation,” and chemotherapeutics cause the dysregulation of transportation and transformation in the spleen, leading to diarrhea. BPHW decoction is a common method for clinical prevention and treatment of toxic side effects after chemotherapy. It can repair damaged intestinal mucosal barrier by regulating gut microbiota and through antiapoptosis, thus effectively reducing the incidence rate of chemotherapeutics-induced vomiting, abnormal defecation, andso on. [10].

In recent years, studies have found that intestinal mucosal damage caused by chemotherapy is closely associated with intestinal mucosal microinflammation [11]. Inflammatory factors can directly damage endothelial cells, increase local vascular permeability, and induce macrophage chemotaxis, synthesis of cyclooxygenase 2 (COX-2), and prostaglandins (PGE2) to further induce pathological alterations including intestinal edema, ulceration, and decreased absorption capacity [12]. In addition, in patients with inflammatory bowel disease, intestinal mucosal permeability is generally increased, as demonstrated by suppressed expression of tight adhesion protein (ZO-1) and adhesion-linked protein (E-cadherin) [13, 14], showing that inflammatory factors play an important role in intestinal mucosal injury.

The triggering agents for intestinal inflammatory factors are complicated. In recent years, with the clarification of the pivotal role of NF-*κ*B in the intestinal inflammation, key molecules that inhibit the TLR/NF-*κ*B pathway have become the research hotspot on the suppression of intestinal inflammation. At rest, NF-*κ*B binds to its inhibitory protein I*κ*B in an inactive state. Physicochemical factors, such as chemotherapy and radiation, can stimulate the activation of pattern recognition receptors (TLRs) on the surface of immune cells for intracellular signal transduction by myeloid differentiation factor 88 (MyD88) followed by phosphorylation and ubiquitination of I*κ*B to activate the NF-*κ*B, thereby causing downstream inflammatory cascades [15].

BPHW decoction consists of *Astragalus*, raw white, clamshell, ginger pinellia, divine comedy, malt, and poria. Our present findings showed that the loss of body weight and the severity of diarrhea were significantly ameliorated in the BPHW decoction than those in the model group, which was consistent with the results of Sijunzi decoction and Shenling powder [16, 17]. In addition, TLR-4/NF-*κ*B-induced intestinal mucosal inflammatory injury is an important factor for causing chemotherapy-associated diarrhea. Here, in this study, we found that the integrity of intestinal villi and the expression of ZO-1 and E-cadherin in the rats of BPHW decoction were ameliorated, while the intestinal expression of TLR-4 and NF-*κ*B of rats, as well as the contents of TNF-*α*, IL-1*β*, and IL-6 was also significantly lower than those in the model group. Moreover, it has also been reported that *Astragalus* [18] and Poria [19] can protect lipopolysaccharide-induced intestinal mucosal injury by reducing the production of inflammatory cytokines, such as IL-1*β*, and TNF-*α* through inhibiting the NF-*κ*B signaling pathway. *Atractylodes* [20] and *Pinellia ternata* [21] can promote cell proliferation and repair by inhibiting the proapoptotic gene (Bax) expression through polyamine-mediated Ca^2+^ signaling pathway. These single-agent pharmacological effects are the fundamental material basis for the intestinal mucosal protection of BPHW decoction, which also confirms that inhibiting TLR-4/NF-*κ*B inflammatory pathway is the mechanism of intestinal mucosal protection of BPHW decoction after chemotherapy.

To sum up, this study confirmed that BPHW decoction significantly inhibited TLR-4/NF-*κ*B activation and the expression of downstream inflammatory cytokines (including TNF-*α*, IL-1*β*, and IL-6) to alleviate 5-Fu-induced intestinal inflammatory injury. However, the Traditional Chinese Medicine compound has relatively diverse active ingredients, and the intestinal inflammatory mechanism is rather complicated, involving various factors, such as immunity, apoptosis, and dysbacteriosis. Therefore, it still deserves further exploration of the cross-effects of various proinflammatory factors, and their relationship with NF-*κ*B pathway as well as the major active components and pathways of Traditional Chinese Medicine compounds.

## Figures and Tables

**Table 1:** Scoring criteria of diarrhea in rats.

Degree of diarrhea	Symptoms	Score
Normal	Normal stool or absent	0
Slight	Slight wet and soft stool	1
Moderate	Wet and unformed stool with moderate perinanal staining of the coat	2
Severe	Watery stool with severe perianal staining of the coat	3

**Table 2:** Primers used for qPCR.

Genes	Forward primer	Reverse primer
GAPDH	5′-GGTGAAGGTCGGTGTGAACG-3′	5′-CTCGCTCCTGGAAGATGGTG-3′
TLR-4	5′-CCTGGCTAGGACTCTGAT-3′	5′-CTTGGTTGAAGAAGGAATGTC-3′
NF-*κ*B	5′-TTACGGGAGATGTGAAGATG-3	5′-ATGATGGCTAAGTGTAGGAC-3′

## Data Availability

The data used to support the findings of this study are included within the article.
